# Use of the C-Reactive Protein (CRP)/Albumin Ratio as a Severity Tool in Acute Pancreatitis: Systematic Review

**DOI:** 10.7759/cureus.29243

**Published:** 2022-09-16

**Authors:** Muhammad Yasir Tarar, Aizaz Khalid, Xin Yin Choo, Sadaf Khurshid, Haitham Tumeh, Karim Muhammad

**Affiliations:** 1 General Surgery, Tameside and Glossop Integrated Care NHS Foundation Trust, Manchester, GBR; 2 General Surgery, St Richards Hospital, Chichester, GBR; 3 Trauma and Orthopaedics, Blackpool Victoria Hospital, Blackpool, GBR; 4 Gynecology, University of Lahore Teaching Hospital, Lahore, PAK

**Keywords:** c-reactive protein (crp), ratio, severity, albumin level, acute pancreatitis (ap)

## Abstract

Acute pancreatitis is one of the most common conditions with high rates of morbidity and mortality. Different scoring systems are used to gauge the severity of this condition, which, in turn, estimates the complications and mortality rates. With the ever-evolving use of the acute-phase reactant protein, C-reactive protein (CRP), and an abundant circulating protein in plasma, albumin, in daily practice, this study aimed to assess the ratio of CRP and albumin for assessing the severity of acute pancreatitis.

A systematic review of the literature was performed using the keywords CRP albumin ratio and acute pancreatitis in the PubMed and Cochrane databases. Studies reporting the use of the ratio of CRP and albumin in acute pancreatitis as well as the outcomes were included in this analysis. The quality of studies was assessed using the MINORS (methodological index for non-randomized studies) assessment tool. In our review, across these three studies, 956 patients with acute pancreatitis were identified and enrolled in studies that examined the relationship between the CRP/Albumin ratio and the severity of acute pancreatitis.

Overall, a positive correlation was found between the CRP/albumin ratio at admission and the development of subsequent severe acute pancreatitis, increased hospital length of stay, and the higher rate of mortality in these studies.

## Introduction and background

Acute pancreatitis is a common condition, with its reported incidence ranging from 4.6 to 100 per 100,000 population in 17 European countries, with gallstones as the most common cause in Southern Europe and alcohol in Eastern Europe [1]. Regardless of the recent advances in the field of medicine, the high acuity of this condition can cause increased mortality and morbidity [2]. There are several grading systems used over the years to assist clinicians in identifying the severity as well as estimating the rate of mortality appropriately. For example, the bedside index for severity in acute pancreatitis (BISAP), acute physiological assessment and chronic health evaluation (APACHE), Glasgow, Atlanta classification, and Ranson scores are all well-known scoring systems used [3,4]. These scoring systems require multiple blood test results and physical parameters to be taken into account, some at different intervals, upon admission, and at 48 hours to allow an accurate calculation to identify its severity.

Recently, the role of C-reactive protein (CRP) has been exemplified in gauging the severity of inflammatory and infective conditions. However, the precise cut-off values of CRP for these conditions remain unknown [5]. It has been reported that CRP levels of more than 210 mg/L in acute pancreatitis differentiate mild and severe cases, with 83% sensitivity and 85% specificity [6]. The levels of albumin, an abundant circulating protein in plasma, can be reduced during sepsis and critical illnesses due to decreased synthesis, increased breakdown, as well as increased vascular permeability, leading to leakage of this protein [7]. This can reflect on its association with the risk of organ failure development and death in acute pancreatitis [8].

There is sparse literature on evaluating the role of the CRP/albumin ratio in acute pancreatitis. This has led to the design of this study as we aim to study the available evidence and to understand the potential of using this ratio routinely as a severity tool in acute pancreatitis.

## Review

**Figure 1:**
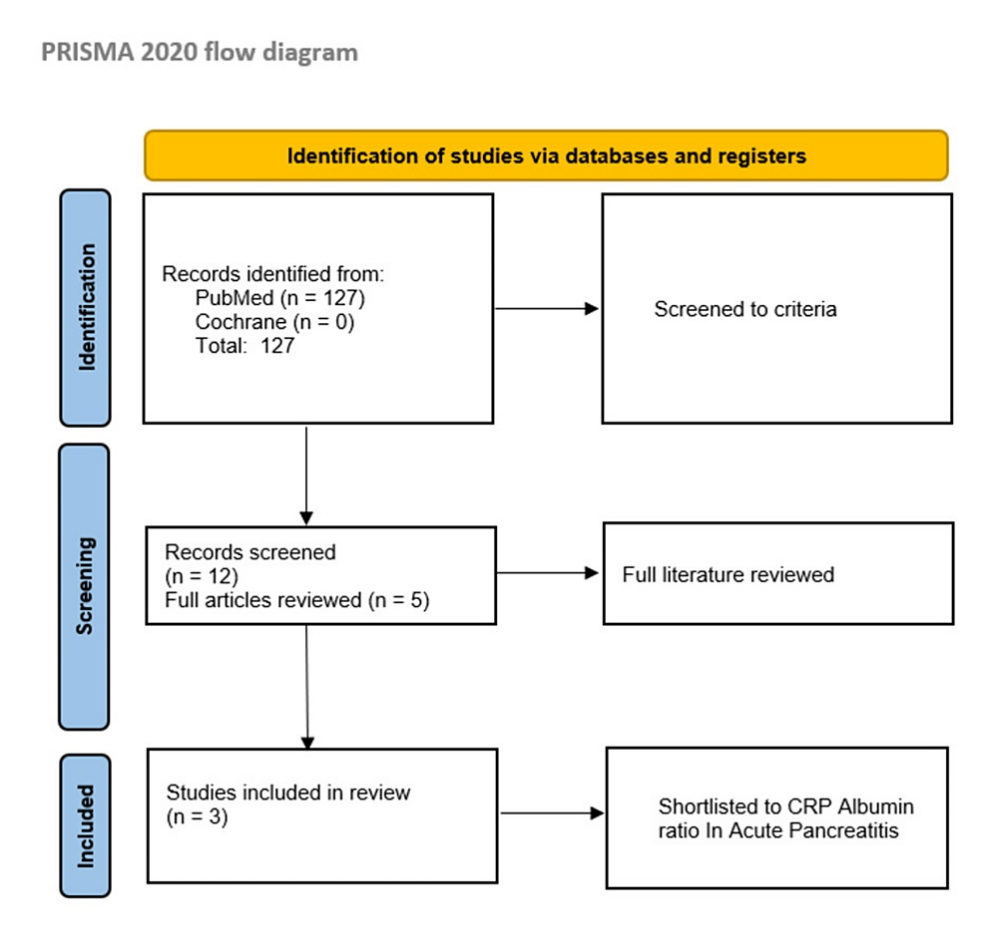
PRISMA study methodology PRISMA: Preferred Reporting Items for Systematic Reviews and Meta-Analyses

A systematic review of the literature was performed using Preferred Reporting Items for Systematic Reviews and Meta-Analyses (PRISMA) guidelines (Figure 1), with the search terms ‘Crp albumin ratio’ and ‘acute pancreatitis’ in PubMed and Cochrane from inception to June 2022. Any study type was initially screened to identify the right study based on the selection criteria. The studies reported disease severity, change in mortality outcomes, and prognosis. The quality of studies was further assessed using the MINORS (methodological index for non-randomized studies) assessment tool (Table 1). The data collected were evaluated on a Microsoft Excel sheet (Microsoft Corporation, Redmond, WA), and tables were made to perform relevant statistical analysis where needed. At present, no protocol has been registered for this study by the authors. Studies with less than five cases were excluded.

**Table 1:** The MINORS assessment tool Articles included scored against MINOR (methodological index for non-randomized studies) The items are scored 0 (not reported), 1 (reported but inadequate), or 2 (reported and adequate). The global ideal score is 16 for non-comparative studies. All these studies are non-comparative studies.

Articles included	Kaplan et al. [9]	Karabuga et al. [10]	Yılmaz et al. [11]	Zhao et al. [12]
Aim clearly stated	2	2	2	2
Inclusion of consecutive patients	2	2	2	2
Prospective collection of data	0	0	0	0
Endpoints appropriate to the aim of the study	2	2	2	2
Unbiased assessment of the study endpoint	0	0	0	0
Follow-up period appropriate to the aim of the study	2	0	0	0
Loss to follow up less than 5%	2	0	0	0
Prospective calculation of the study size	0	0	0	0
Total score (out of 16)	8	6	6	6

In our review, across three studies, we identified 956 patients with acute pancreatitis that were enrolled in studies that examined the relationship of the CRP/albumin ratio with the severity of acute pancreatitis (Table 2). Four-hundred seventy-eight (478; 50%) of these patients were males and 478 (50%) were females. Seven-hundred fifty (755; 79%) of the cases were attributed to having non-severe pancreatitis while 201 (215) had severe pancreatitis. All three studies used recognized scoring systems as the standard against which the performance of the CRP/albumin ratio was evaluated; these scoring systems included the Ranson, Atlanta, and BISAP scoring systems. A positive correlation was found between the CRP/albumin ratio at admission and the development of subsequent severe acute pancreatitis in these studies (Tables 2-3) [9-11].

**Table 2:** Study demographics

Study Name	Year Published	Retro/Prospective	Study type (Case series, cohort, RCT)	Age, mean (range)/SD	Sex, M:F	Severity of cases by number
Kaplan et al. [9]	2017	Retrospective	Cohort	61.9 ± 18.0	72:120	Ranson “0”: 29(15.1%); “1”: 36 (18.8%); “2”: 44 (22.9%); “3”: 31 (16.1%); “4”: 17 (8.9%); “5”: 25 (13%); Atlanta “mild”: 127 (66.1) “moderately severe”: 36 (18.8)); “severe”: 29 (15.1%)
Karabuga et al. [10]	2022	Retrospective	Cohort study	50.19 ± 16.01	247:253	BISAP <3, mild AP: 388 (77.6%); BISAP≥ 3, severe AP: 112 (22.4%)
Yılmaz et al. [11]	2018	Retrospective	Cohort study	59.97 (21-95) ±17.47	105:159	Defined as the Ranson score >3, N=60 (22.8%)
Zhao et al. [12]	2020	Retrospective	Cohort study	49.88 ± 13.94	98:42	Defined using the Atlanta score 46 (32.86%)

**Table 3:** Assessment of ratio as a tool CRP: C-reactive protein; NLR: neutrophil-to-lymphocyte ratio

Study Name	Year conducted	Number of patients	CRP/albumin ratio values, mean mg/L (range)	Mortality	Complications	Follow-up, median	Study’s recommendation
Kaplan et al. [9]	Jan 2002 - June 2015	192	The ratio of 16.28 had a 19.3x change in death	38 (19.8%)	Acute renal failure: 17 (8.9%); Abscess: 8 (4.2%); Sepsis: 10 (5.2%); Pseudocyst: 9 (4.7%); Ascites: 3 (1.6%); Haematoma: 5 (2.6%); Cholangitis: 6 (3.1%); Oedematous: 153 (79.7%); Necrotizing pancreatitis: 38 (19.8%)	63 months (1-126)	CRP/albumin ratio could be used to predict prognosis in patients with acute pancreatitis.
Karabuga et al. [10]	Feb 2019 – March 2020	500	0.0181 ± 0.00232; Median: 0.00083	Mild AP: 2 out of 388 (0.52%); Severe AP: 21 out of 112 (18.75%); Total: 23 out of 500 (4.6%)	N/A	N/A	NLR and CRP/albumin values were found most reliable in determining the severity of acute pancreatitis. Recommends usability of these inexpensive parameters.
Yılmaz et al. [11]	Jan 2014 – Nov 2017	264	19.16 (0.05-114.94) ± 26.09	0	22 (8.3%)	N/A	Highlight the CRP/albumin ratio promising a potential marker for use in determining prognosis in acute pancreatitis cases
Zhao et al. [12]	Jan 2008 – Nov 2019	140	Single-operation: 2.90±3.02; Re-operation: 4.63±2.8; Survival: 3.32 ±2.88	16 (11.43%)	90 (64.29%)	N/A	The creatinine/albumin showed better performance than CRP/albumin

The use of the inflammatory ratio involving CRP and prealbumin (PALB) for prognostic purposes can be traced back to 1998 when Pinilla et al. established a strong correlation between CRP/PALB with severe organ dysfunction in patients with sepsis [13]. The first study to use CRP/albumin to predict patient outcomes was published in 2009 when the efficacy of this marker was compared to the modified early warning score (MEWS) by Fairclough et al. [14]. They found that for patients that were admitted to the acute medical unit, MEWS outperformed the CRP/albumin ratio but mortality rose from 5% to 25% if this ratio increased from <2 to >4. Ranzani et al. also found that the CRP/albumin ratio is an independent risk factor of 90-day mortality in patients with sepsis (Table 3) [15].

CRP is a positive acute phase reactant that is produced by hepatocytes in response to systemic inflammatory markers such as interleukin 6 (IL-6). Albumin, on the other hand, is a negative acute phase reactant that decreases due to such signals. Hypoalbuminemia has been shown to be a potent, dose-dependent independent predictor of poor outcomes [16]. The use of this ratio to predict severity in acute pancreatitis is very promising due to the pathophysiology of this disease. Acute pancreatitis triggers local and systemic inflammatory responses, especially in its severe form, which would inevitably affect these hepatic markers.

The role of inflammation in neoplastic disease has led to the use of the CRP/albumin ratio to detect outcomes in patients with cancer. Kinoshita et al. first studied this relationship and found the ratio to predict tumor progression and decreased liver functional reserve in patients with hepatocellular carcinoma (HCC) [17]. A cut-off value of >0.037 was deemed an early sign of poor outcomes in HCC. Zhou et al. studied the role of CRP/albumin in patients with small cell lung carcinoma and found that patients with a ratio of more than 0.441 had 1.34 times the risk of death than those less than 0.441, thus establishing this ratio as an independent prognostic indicator for patients with small cell lung carcinoma [18]. More studies also established the role of the CRP/Albumin ratio as a prognostic marker in esophageal squamous cell CA and colorectal carcinoma (Table 3) [19,20].

The first assessment of this ratio's correlation with the severity of pancreatitis was done in 2015 by Kaplan et al. [9]. They found that the CRP/albumin ratio positively correlated to hospital length of stay (p<0.001), Atlanta classification of severity of disease, and Ranson scoring. The study also found the ratio to be an independent risk factor for mortality. A CRP/albumin ratio of >16.28 was found to be associated with mortality, with 92.1% sensitivity and 58% specificity. They found that if the ratio was greater than 16.28, it corresponded to 19.271 times higher mortality than if it was lower than 16.28. Furthermore, median survival as noted by the area under the curve (AUC) with a ratio of >16.28 was noted to be 55 months (Table 3).

The relationship between the CRP/albumin ratio and severe acute pancreatitis was also studied by Yilmaz et al. [10]. They used Ranson scoring and found the ratio to predict severity with 66% sensitivity and 90% specificity if the ratio was >8.51. They also found that the ratio predicted increased hospital and ICU length of stay. Similar conclusions were drawn by Karabuga et al., who analyzed this relationship of severity using the BISAP score [11]. They found that for a cut-off of 0.0015, the ratio was 71.43% sensitive and 70.88% specific for predicting severe acute pancreatitis.

The contrast to the cut-offs between these two studies has been suggested to be due to different threshold values of the hepatic parameters and different scoring systems. Zhao et al. studied the prognostic values of the CRP/albumin ratio in patients with acute pancreatitis that needed surgical debridement [20]. They found that this ratio was significantly associated with higher chances of re-operation after initial debridement (p<0.05), as well as prolonged ICU length of stay ( p = 0.003).

Over the last two decades, the CRP/albumin ratio has emerged as a strong prognostic indicator in several areas of medicine. Our studies reviewed available studies on this ratio, which found an overall positive correlation between the CRP/albumin ratio at admission, as well as the development of severe acute pancreatitis. The main utility of this ratio lies in the fact that these parameters are readily assessable and can be calculated regularly and easily. It is simple and not technical, which makes it an invaluable asset for any healthcare assessment. Early patient stratification according to the potential severity is of paramount importance in acute pancreatitis; hence, more studies are needed to assess the utility of the CRP/albumin ratio as a prognostic tool.

## Conclusions

Our systematic review has shown a positive correlation was found between the CRP/albumin ratio at admission and the development of subsequent severe acute pancreatitis, increased hospital length of stay, and higher rate of mortality in these studies. We believe the CRP/albumin ratio is easy to calculate and gauge the severity of acute pancreatitis.

## Appendices

 The PRISMA checklist (Table 4) was completed [21].

**Table 4:** PRISMA checklist PRISMA: Preferred Reporting Items for Systematic Reviews and Meta-Analyses PRISMA checklist [21]

Section and Topic	Item #	Checklist item	Location where item is reported
TITLE			
Title	1	Identify the report as a systematic review.	1
ABSTRACT			
Abstract	2	See the PRISMA 2020 for Abstracts checklist.	1
INTRODUCTION			
Rationale	3	Describe the rationale for the review in the context of existing knowledge.	1, 2
Objectives	4	Provide an explicit statement of the objective(s) or question(s) the review addresses.	1, 2
METHODS			
Eligibility criteria	5	Specify the inclusion and exclusion criteria for the review and how studies were grouped for the syntheses.	3
Information sources	6	Specify all databases, registers, websites, organisations, reference lists and other sources searched or consulted to identify studies. Specify the date when each source was last searched or consulted.	3
Search strategy	7	Present the full search strategies for all databases, registers and websites, including any filters and limits used.	3
Selection process	8	Specify the methods used to decide whether a study met the inclusion criteria of the review, including how many reviewers screened each record and each report retrieved, whether they worked independently, and if applicable, details of automation tools used in the process.	3
Data collection process	9	Specify the methods used to collect data from reports, including how many reviewers collected data from each report, whether they worked independently, any processes for obtaining or confirming data from study investigators, and if applicable, details of automation tools used in the process.	3
Data items	10a	List and define all outcomes for which data were sought. Specify whether all results that were compatible with each outcome domain in each study were sought (e.g. for all measures, time points, analyses), and if not, the methods used to decide which results to collect.	3
	10b	List and define all other variables for which data were sought (e.g. participant and intervention characteristics, funding sources). Describe any assumptions made about any missing or unclear information.	3
Study risk of bias assessment	11	Specify the methods used to assess risk of bias in the included studies, including details of the tool(s) used, how many reviewers assessed each study and whether they worked independently, and if applicable, details of automation tools used in the process.	Table 3, 3
Effect measures	12	Specify for each outcome the effect measure(s) (e.g. risk ratio, mean difference) used in the synthesis or presentation of results.	Table 2
Synthesis methods	13a	Describe the processes used to decide which studies were eligible for each synthesis (e.g. tabulating the study intervention characteristics and comparing against the planned groups for each synthesis (item #5)).	Table 1
	13b	Describe any methods required to prepare the data for presentation or synthesis, such as handling of missing summary statistics, or data conversions.	3, Table 1
	13c	Describe any methods used to tabulate or visually display results of individual studies and syntheses.	Table 1,2
	13d	Describe any methods used to synthesize results and provide a rationale for the choice(s). If meta-analysis was performed, describe the model(s), method(s) to identify the presence and extent of statistical heterogeneity, and software package(s) used.	n/a
	13e	Describe any methods used to explore possible causes of heterogeneity among study results (e.g. subgroup analysis, meta-regression).	n/a
	13f	Describe any sensitivity analyses conducted to assess robustness of the synthesized results.	n/a
Reporting bias assessment	14	Describe any methods used to assess risk of bias due to missing results in a synthesis (arising from reporting biases).	Table 3
Certainty assessment	15	Describe any methods used to assess certainty (or confidence) in the body of evidence for an outcome.	Table 3
RESULTS			
Study selection	16a	Describe the results of the search and selection process, from the number of records identified in the search to the number of studies included in the review, ideally using a flow diagram.	Table 1, 2
	16b	Cite studies that might appear to meet the inclusion criteria, but which were excluded, and explain why they were excluded.	Table 1, 2
Study characteristics	17	Cite each included study and present its characteristics.	Table 1, 2
Risk of bias in studies	18	Present assessments of risk of bias for each included study.	Table 3
Results of individual studies	19	For all outcomes, present, for each study: (a) summary statistics for each group (where appropriate) and (b) an effect estimate and its precision (e.g. confidence/credible interval), ideally using structured tables or plots.	Table 2, Review
Results of syntheses	20a	For each synthesis, briefly summarise the characteristics and risk of bias among contributing studies.	Review
	20b	Present results of all statistical syntheses conducted. If meta-analysis was done, present for each the summary estimate and its precision (e.g. confidence/credible interval) and measures of statistical heterogeneity. If comparing groups, describe the direction of the effect.	Review
	20c	Present results of all investigations of possible causes of heterogeneity among study results.	Review
	20d	Present results of all sensitivity analyses conducted to assess the robustness of the synthesized results.	Review
Reporting biases	21	Present assessments of risk of bias due to missing results (arising from reporting biases) for each synthesis assessed.	Table 3
Certainty of evidence	22	Present assessments of certainty (or confidence) in the body of evidence for each outcome assessed.	Table 3
DISCUSSION			
Discussion	23a	Provide a general interpretation of the results in the context of other evidence.	Review
	23b	Discuss any limitations of the evidence included in the review.	Review
	23c	Discuss any limitations of the review processes used.	Review
	23d	Discuss implications of the results for practice, policy, and future research.	Review
OTHER INFORMATION			
Registration and protocol	24a	Provide registration information for the review, including register name and registration number, or state that the review was not registered.	Not yet
	24b	Indicate where the review protocol can be accessed, or state that a protocol was not prepared.	N/a
	24c	Describe and explain any amendments to information provided at registration or in the protocol.	n/a
Support	25	Describe sources of financial or non-financial support for the review, and the role of the funders or sponsors in the review.	Cureus site
Competing interests	26	Declare any competing interests of review authors.	Appendix
Availability of data, code and other materials	27	Report which of the following are publicly available and where they can be found: template data collection forms; data extracted from included studies; data used for all analyses; analytic code; any other materials used in the review.	n/a
